# Abdominal fascia closure following elective midline laparotomy: a surgical experience at a tertiary care hospital in Tanzania

**DOI:** 10.1186/s13104-015-1243-4

**Published:** 2015-06-30

**Authors:** Phillipo L Chalya, Anthony N Massinde, Albert Kihunrwa, Joseph B Mabula

**Affiliations:** Department of Surgery, Catholic University of Health and Allied Sciences-Bugando, Mwanza, Tanzania; Department of Obstetrics and Gynaecology, Catholic University of Health and Allied Sciences-Bugando, Mwanza, Tanzania

**Keywords:** Elective midline laparotomy, Abdominal fascial closure, Practices, Postoperative complications, Tanzania

## Abstract

**Background:**

The optimal strategy of abdominal wall closure after midline laparotomy has remained an issue of ongoing debate. This study was undertaken to describe our own experiences with abdominal fascial closure following elective midline laparotomy and compare with what is described in literature.

**Methods:**

This was a descriptive prospective study of patients who underwent elective midline laparotomy at Bugando Medical Centre between March 2009 and February 2014.

**Results:**

A total of 872 patients (M:F = 2.8:1) were studied. The median age was 38 years. The fascia closure was performed with a continuous and interrupted sutures in 804 (92.2%) and 68 (7.8%) patients, respectively. Mass closure and layered closure were performed in 842 (96.6%) and 30 (3.4%) patients, respectively. Monofilament sutures were applied for fascia closure in 366 (42.0%) patients, multifilament sutures in 506 (58.0%) patients. Non-absorbable sutures were chosen in 304 (34.9%) patients, slowly absorbable sutures in 506 (58.0%), and moderately absorbable sutures in 62 (7.1%) patients. Sutures used for fascial closure were vicryl 464 (53.2%), nylon 250 (28.7%), prolene 62 (7.1%), PDSII 54 (6.2%) and silk 42 (4.8%). Sutures with the strength of 0 were used in 214 (24.4%) patients, with strength of 1 in 524 (60.1%) patients, and with strength of 2 in 134 (15.4%) patients. The mean time required for massive closure of the midline incision was 8.20 ± 6.12 min whereas in layered closure, the mean time required for closure was 12.22 ± 7.11 min and this was statistically significant (p = 0.002). Mass closure was significantly associated with low incidence of wound dehiscence and incisional hernia (p < 0.001). Continuous suture was significantly associated with low incidence of wound dehiscence and incisional hernia as compared to interrupted suture (p < 0.001). Non-absorbable sutures were significantly associated with increased incidence of persistent wound pain and stitch sinus as compared to absorbable sutures (p < 0.001). The use of monofilament sutures was insignificantly associated with low incidence of surgical site infection as compared to multifilament sutures (p = 0.051). Prolene was significantly associated with persistent wound pain as compared to vicryl (p = 0.017).

**Conclusion:**

Continuous mass closure with vicryl is commonly used for abdominal fascial closure following elective midline laparotomy in our setting and gives satisfactory results.

## Background

Exploratory laparotomy remains one of the common operations across the surgical disciplines. As such, the systematic and safe closure of such a laparotomy wound is the key to reduce the postoperative morbidity like wound pain, wound infection and incisional hernia [1]. This, in turn, may lead to early discharge from the hospital, early return to activities and has a potential of eventually saving the overall cost of the procedure.

A midline incision is frequently used in abdominal surgery. It provides a relatively quick and wide access to the abdominal cavity and can be made with minimal damage to muscles, nerves and blood supply as these structures do not cross the midline [2–6].

Techniques for closure of the midline abdominal incision have varied over time with better understanding of the physiology and engineering of closure of the abdominal wall and improvement in materials of surgical suture. The ideal wound closure provides strength and barrier to infection. To achieve that goal closure should be fast, efficient, performed without tension/ischaemia, comfortable to the patient, technically easier to surgeon and aesthetic. Hence, one should follow the principles of wound closure [6].

The optimal strategy of abdominal wall closure after midline laparotomy has remained an issue of ongoing debate. To date, various randomized clinical trials and meta-analyses on abdominal wall closure strategies after midline laparotomy have been published with heterogeneous results. A recent meta-analysis identified several randomized clinical trials on techniques and materials of abdominal wall closure after elective laparotomy [4]. However, despite these meta-analyses and randomized clinical trials, the optimal technique and material for abdominal fascia closure after midline laparotomy remains inconclusive as a result, abdominal fascia closure is performed according to the surgeon’s individual preference rather than according to evidence-based data. Lack of guidelines for abdominal fascial closure leaves surgeons uncertain about the optimal technique and suture material to be used worldwide. Thus, practices of abdominal fascial closure differs greatly from one centre to another, differences also exists even among surgeons themselves in the same centre [4, 5]. Complications arising following abdominal fascial closure are fairly common especially in resource limited countries like Tanzania [7]. Hence, it was important to understand the practices and associated complications of fascial closure at Bugando Medical Centre in order to improve surgical outcomes. This study was undertaken to describe our experiences with abdominal fascial closure following elective midline laparotomy. The aim of this study was to determine the practices and associated complications of abdominal fascial closure following elective midline laparotomy at closure at Bugando Medical Centre, tertiary care hospital in northwestern Tanzania.

## Methods

### Study design and setting

This was a descriptive prospective study of patients who underwent elective midline laparotomy in general surgical, urological and gynecological wards of Bugando Medical Centre over a period of 5 years from March 2009 to February 2014. Bugando Medical Center is a tertiary and teaching hospital for the Catholic University of Health and Allied Sciences-Bugando (CUHAS-Bugando) in the Lake and Western zones of the United Republic of Tanzania. It is situated along the shores of Lake Victoria in Mwanza City. It has 1,000 beds and serves as a referral center for tertiary specialist care for a catchment population of approximately 13 million people from neighboring regions.

### Study population

Patients undergoing primary elective midline laparotomy in general surgery, urology and gynecology units during the study period and those who consented to participate in the study were included. Patients with previous abdominal incisions, patients who were operated by pfannenstiel incision, patients with advanced malignancies (inoperable malignancies), patients whose information on the technique and/or suture material used was not known, patients who lost to follow up and those who died during follow up period were excluded from the study. Patients aged 10 years and younger were also excluded from the study as these are admitted in the paediatric surgical wards.

All patients scheduled for primary elective midline laparotomy underwent preoperative care according to Bugando Medical Centre protocol. Recruitment of patients to participate in the study was done in the surgical, urological and gynaecological wards of Bugando Medical Centre. All patients included in the study were pre-operatively evaluated by means of history taking and physical examination. Relevant investigations were performed to establish diagnosis and to assess general fitness for surgery. Complete blood count, blood sugar, blood urea and where required ECG and chest X-ray were done. In addition ultrasound abdomen, CT scan, intravenous urography, gastro-duodenoscopy, or contrast studies of the gut were performed when indicated. Pre-operative antibiotic prophylaxis was given at the time of induction of anesthesia and included single dose cephalosporin, gentamicin and metronidazole.

Intraoperatively, all patients under general anesthesia were subjected to exploratory laparotomy through midline incision. The operations were performed either by a consultant surgeon or a senior resident under the direct supervision of a consultant surgeon. Operation notes were inspected for the type of surgery done, technique of fascial closure and suture material used. In case these were not documented properly, communication with the surgical team was made to accomplish this task. Post-operatively patients were kept nil orally till return of bowl sounds and at that time nasogastric tubes were removed depending on the volume of the nasogastric tube drainage. Wound incision was examined usually on 5th postoperative day. However it was seen earlier in cases when dressing became soaked or patient developed fever or tachycardia and no other source of fever or tachycardia was found. Postoperatively these patients were assessed for complications related to abdominal incision (e.g. surgical site infection, persistent wound pain, stitch sinus formation, wound dehiscence, incisional hernia, keloids etc.). Patients were discharged from hospital once oral feeding was tolerated well and patients became freely mobile. Patients were asked to attend our clinic at 1, 3 and 6 months or at any time if there was development of a problem from the scar; for those who were not able to attend clinic more than once, on phone interview was conducted. All study subjects were followed up for a minimum of 2 years for development of incisional hernia.

Patients’ data was recorded on a pre-designed questionnaire. Included in the questionnaire were age and sex of the patient, type of surgical procedure performed, type of fascia closure (massive/layered), fascial suture technique (continuous/interrupted), suture absorption (absorbable/non-absorbable), suture material (monofilament/multifilament), suture type (prolene/nylon/silk/vicryl/PSD II) and postoperative wound complications (surgical site infections, wound dehiscence, incisional hernia, stitch sinus formation, persistent wound pain and keloids formation).

### Definition of terms

*Surgical site infection* was diagnosed if any one of the following criteria was fulfilled: purulent drainage from the incision, organisms isolated from an aseptically obtained culture of fluid or tissue from the superficial incision, at least one of the following signs or symptoms of infection: pain or tenderness, localized swelling, redness, or heat. *Wound dehiscence* was defined as a complete disruption of the wound with or without evisceration of abdominal content demanding emergent reoperation. *Incisional hernia* was defined clinically as a palpable incisional fascial defect ≥2 cm in diameter, or visible bulge in the laparotomy incision) within 2 years of operation. Patients who had wound pain which was causing any degree of limitation to activities beyond one month were considered to have *persistent wound pain*.

### Statistical data analysis

The statistical analysis was performed using statistical package for social sciences (SPSS) version 17.0 for Windows (SPSS, Chicago IL, USA). The median (+IQR) and ranges were calculated for continuous variables whereas proportions and frequency tables were used to summarize categorical variables. Chi square (χ2) test were used to test for the significance of association between the independent and dependent (outcome) variables in the categorical variables. The level of significance was considered as P < 0.05. Multivariate logistic regression analysis was used to determine predictor variables that predict the postoperative complications.

### Ethical consideration

Ethical approval to conduct the study was obtained from the CUHAS/BMC joint institutional ethic review committee before the commencement of the study. Patients who met the inclusion criteria were requested to sign a written informed consent before being enrolled into the study.

## Results

During the period of study, a total of 936 patients were admitted to our centre and underwent laparotomy for various abdominal conditions. Of these, 64 patients were excluded from the study due to failure to meet the inclusion criteria. Thus, 872 were enrolled into the study. Six hundred and forty-two (73.6%) were males and 230 (26.4%) females, with a male to female ratio of 2.8: 1. The age of patients at presentation ranged from 12 to 86 years with a median age of 38 years (IQR = 36–40 years). The peak age incidence was in the age group of 31–40 years accounting for 382 (43.8%) patients.

All patients in this study underwent laparotomy for various abdominal conditions through midline incision. Out of the 872 laparotomies, extended midline incision was made in 644 (73.9%) patients, lower (sub-umbilical) midline incisions (SUMI) in 214 (24.5%) patients and upper midline incisions in 14 (1.6%) patients. Table 1 shows types of surgical procedures performed.

**Table 1:** Types of surgical procedures performed (n = 872)

Specialty	Type of surgical procedures	Number of patients	Percentages
General surgery (n = 462, 53.0%)	Tumor resection	206	44.5
	Gastro-jejunostomy	61	13.2
	Gastrectomy	50	10.8
	Splenectomy	48	10.4
	Cholecystojejunostomy	40	8.7
	Gastro-cystostomy for pancreatic pseudocyst	14	3.0
	Sigmoid colectomy for redundant sigmoid colon	10	2.2
	Others	33	7.1
Urology (n = 216, 24.8%)	Open prostatectomy	96	44.4
	Nephrectomy	36	16.7
	Nephrolithotomy	32	14.8
	Ureteric implantation	32	14.8
	Others	20	9.3
Gynecological (n = 194, 22.2%)	TAH	98	50.5
	Tubo-ovarian mass excision	62	32.0
	Others	34	17.5

Fascia closure for midline incisions was performed with a continuous suture in 804 (92.2%) patients and interrupted sutures in 68 (7.8%) patients, respectively. Mass closure (single-layer) and layered closure were performed in 842 (96.6%) and 30 (3.4%) patients, respectively. Monofilament sutures were applied for fascia closure in 366 (42.0%) patients, braided (multifilament) sutures in 506 (58.0%) patients. Non-absorbable sutures were chosen in 304 (34.9%) patients, slowly absorbable sutures in 506 (58.0%) patients, and moderately absorbable sutures in 62 (7.1%) patients. Sutures used for fascial closure were vicryl 464 (53.2%), nylon 250 (28.7%), prolene 62 (7.1%), PDSII 54 (6.2%) and silk 42 (4.8%). Sutures with the strength of 0 were used in 214(24.4%) patients, with strength of 1 in 524 (60.1%) patients, and with strength of 2 in 134 (15.4%) patients.

The overall mean time required for abdominal fascial closure of the midline incision was 11.30 ± 4.18 min (range 5.23–16.48 min). The mean time required for massive closure of the midline incision was 8.20 ± 6.12 min whereas in layered closure, the mean time required for closure was 12.22 ± 7.11 min. The difference between the two groups with respect to the mean time required for closure was statistically significant (p = 0.002).

**Figure 1:**
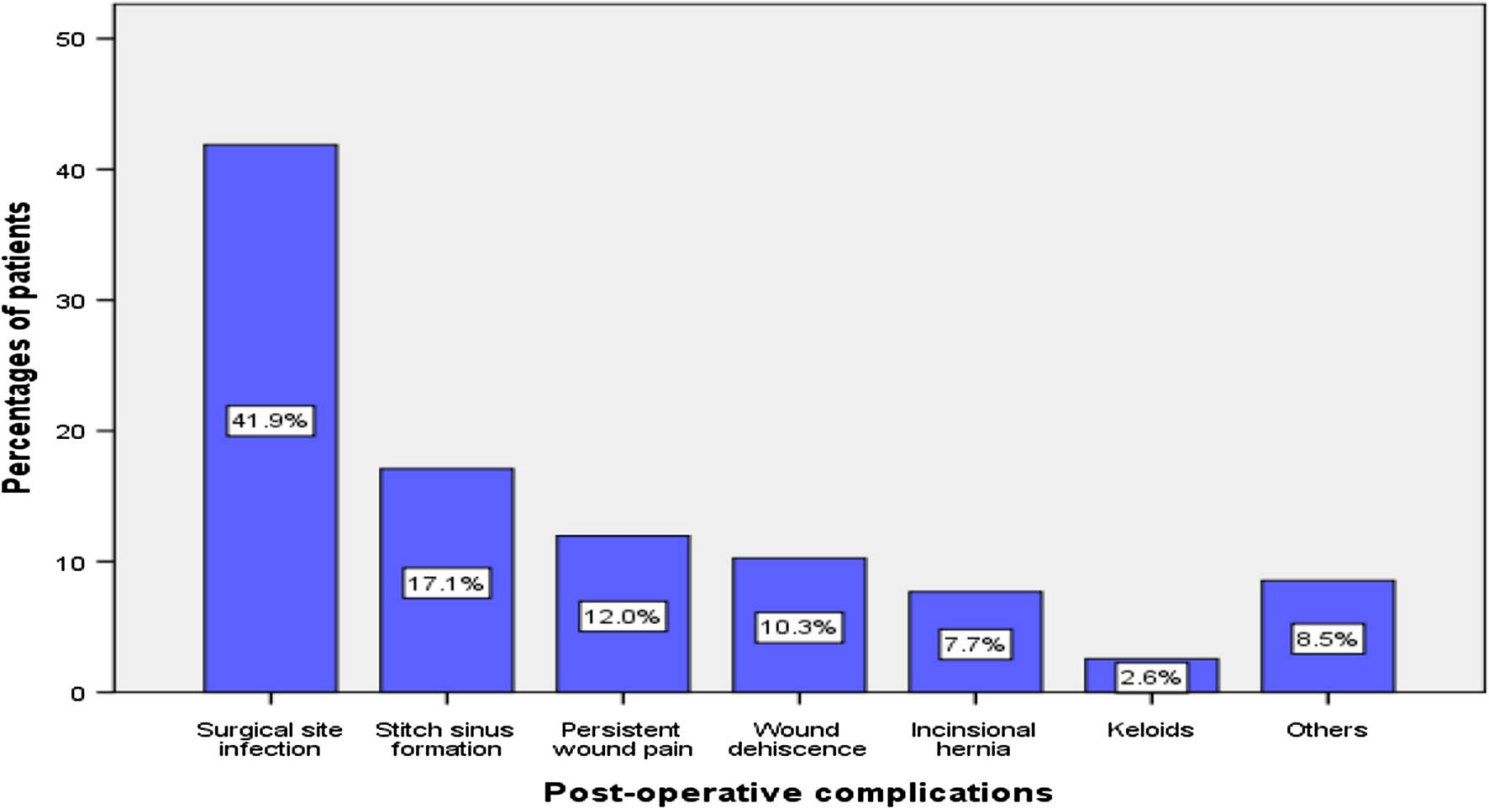
Post-operative complications.

A total of 234 post-operative abdominal complications were recorded in 165 patients giving a complication rate of 18.9%. Of these, surgical site infection was the most common post-operative complication accounting for 41.9% of cases (Figure 1).

Mass closure was significantly associated with low incidence of wound dehiscence (p = 0.011) and incisional hernia (p = 0.004) as compared to layered closure. Continuous suture was significantly associated with low incidence of wound dehiscence (p = 0.003) and incisional hernia as compared to interrupted suture (p = 0.013). Non-absorbable sutures were found to be significantly associated with increased incidence of persistent wound pain (p = 0.012) and stitch sinus (p = 0.023) as compared to slowly absorbable sutures. There was no significantly association between non-absorbable sutures and slowly absorbable sutures with respect to incidence of wound dehiscence (p = 0.031) and incisional hernia (p = 0.024). The use of monofilament sutures was associated with low incidence of surgical site infection as compared to multifilament sutures. However, this association was not found to be statistically significant (p = 0.051). There was no significantly association between multifilament and monofilament sutures with respect to persistent wound pain (p = 0.511) and keloid formation (p = 0.964). Prolene was significantly associated with persistent wound pain as compared to vickyl (p = 0.017). None of our patients in the PDSII group developed postoperative complications. Tables 2, 3, 4, 5 and 6 shows factors associated with postoperative complications as shown by univariate and multivariate analyses results.

**Table 2:** Factors associated with surgical site infection as shown by univariate and multivariate analyses results (N = 98)

Independent variables	Surgical site infection		Univariate analysis		Multivariate analysis	
	Yes (N/%)	No (N/%)	OR (95% CI)	*p* value	OR (95% CI)	p value
Type of closure						
Mass closure	86 (42.2)	118 (57.8)				
Layered closure	12 (40.0)	18 (60.0)	2.1(0.2–3.4)	0.985		
Fascia suture technique						
Continuous	70 (42.2)	96 (57.8)				
Interrupted	28 (41.2)	19 (58.8)	0.3 (0.1–2.2)	0.134		
Suture absorption						
Non-absorbable	34 (42.5)	46 (57.5)				
Absorbable	64 (45.1)	78 (54.9)	1.3 (0.3–2.5)	0.076		
Suture material						
Monofilament	24 (36.4)	42 (63.6)				
Multifilament	74 (44.0)	94 (56.0)	2.1 (1.1–3.2)	0.011	1.4 (0.4–2.6)	0.051
Suture type						
Prolene/nylon	44 (42.3)	60 (57.7)				
Vicryl	54 (41.5)	76 (58.5)	1.6 (0.5–2.7)	0.432

**Table 3:** Factors associated with wound dehiscence as shown by univariate and multivariate analyses results (N = 24)

Independent variables	Wound dehiscence		Univariate analysis		Multivariate analysis	
	Yes (N/%)	No (N/%)	OR (95% CI)	p value	OR (95% CI)	p value
Type of closure						
Massive closure	14 (6.9)	190 (93.1)				
Layered closure	10 (33.3)	20 (66.7)	3.8 (2.2–7.4)	0.002	5.1 (3.2–8.4)	0.011
Fascia suture technique						
Continuous	9 (5.4)	157 (94.6)				
Interrupted	15 (22.1)	53 (77.9)	6.1 (2.9–9.4)	0.001	3.2 (1.7–6.0)	0.003
Suture absorption						
Non-absorbable	19 (9.3)	185 (90.7)				
Absorbable	5 (7.6)	63 (92.6)	1.7 (0.4–2.1)	0.128		
Suture material						
Monofilament	10 (10.6)	84 (89.4)				
Multifilament	14 (12.7)	96 (87.3)	2.8 (0.4–3.9)	0.481		
Suture type						
Prolene/nylon	11 (11.7)	83 (88.3)				
Vicryl	13 (11.8)	97 (88.2)	1.9 (0.8–2.9)	0.945

**Table 4:** Factors associated with incisional hernia as shown by univariate and multivariate analyses results (N = 18)

Independent variables	Incisional hernia		Univariate analysis		Multivariate analysis	
	Yes (N/%)	No (N/%)	OR (95% CI)	p value	OR (95% CI)	p value
Type of closure						
Massive closure	9 (4.4)	195 (95.6)				
Layered closure	9 (30.0)	21 (70.0)	1.7 (2.8–7.1)	0.011	1.1 (1.0–8.1)	0.004
Fascia suture technique						
Continuous	7 (3.4)	197 (96.6)				
Interrupted	11 (36.7)	19 (63.3)	6.4 (2.6–9.0)	0.014	4.2 (3.2–7.8)	0.013
Suture absorption						
Non-absorbable	16(7.8)	188 (92.2)				
Absorbable	2 (6.7)	28 (93.3)	1.1 (0.8–2.9)	0.192		
Suture material						
Monofilament	15 (7.4)	189 (92.6)				
Multifilament	3 (10.0)	27 (90.0)	2.3 (0.9–5.6)	0.992		
Suture type						
Prolene/nylon	14 (6.9)	190 (93.1)				
Vicryl	4 (13.3)	26 (86.7)	1.9 (0.8–4.7)	0.235

**Table 5:** Factors associated with stitch sinus formation as shown by univariate and multivariate analyses results (N = 40)

Independent variables	Stitch sinus formation		Univariate analysis		Multivariate analysis	
	Yes (N/%)	No (N/%)	OR (95% CI)	p value	OR (95% CI)	p value
Type of closure						
Massive closure	35 (16.7)	169 (83.3)				
Layered closure	5 (17.2)	25 (82.8)	1.1 (0.8–2.1)	0.611		
Fascia suture technique						
Continuous	32 (15.7)	172 (84.3)				
Interrupted	8 (26.7)	22 (73.3)	1.4 (0.6–4.0)	0.814		
Suture absorption						
Non-absorbable	24 (23.1)	80 (76.9)				
Absorbable	16 (12.3)	114 (87.7)	4.5 (3.1–8.9)	0.024	1.3 (1.1–4.6)	0.023
Suture material						
Monofilament	18 (17.3)	86 (82.7)				
Multifilament	22 (16.4)	112 (83.6)	1.2 (0.9–3.6)	0.452		
Suture type						
Prolene/nylon	28 (28.0)	72 (72.0)				
Vicryl	12 (8.9)	122 (91.1)	3.9 (2.8–4.6)	0.032

**Table 6:** Factors associated with persistent wound pain as shown by univariate and multivariate analyses results (n = 28)

Independent variables	Persistent wound pain		Univariate analysis		Multivariate analysis	
	Yes (N/%)	No (N/%)	OR (95% CI)	p value	OR (95% CI)	p value
Type of closure						
Massive closure	24 (11.8)	180 (88.2)				
Layered closure	4 (13.3)	26 (86.7)	3.1 (0.9–6.6)	0.392		
Fascia suture technique						
Continuous	23 (11.3)	181 (88.7)				
Interrupted	5 (16.7)	25 (83.3)	0.2 (0.6–2.1)	0.459		
Suture absorption						
Non-absorbable	27 (13.2)	177 (86.8)				
Absorbable	1 (3.3)	29 (96.7)	4.8 (2.6–8.5)	0.022	2.9 (2.1–8.8)	0.012
Suture material						
Monofilament	26 (12.1)	188 (87.9)				
Multifilament	2 (10.0)	18 (90.0)	1.5 (0.1–1.9)	0.764		
Suture type						
Prolene/nylon	24 (20.0)	96 (80.0)				
Vicryl	4 (3.5)	110 (96.5)	2.9 (1.9–9.6)	0.002	5.1 (2.8–9.5)	0.017

## Discussion

Elective midline exploratory laparotomy and its closure is a frequent performed procedure in any surgical unit worldwide and secure closure of a laparotomy incision remains an important aspect of any abdominal operation with the aim to avoid the postoperative morbidity and hasten the patient’s recovery [1]. In this study, the majorities of patients were in the third and fourth decades of life and showed male predominance. Similar age and gender distribution was reported by other authors [4–7]. Komba [7] in Dar Es Salaam, Tanzania reported larger number of females reflect larger numbers of gynaecological operations in his study. We could not establish the reasons for that age and gender differences in our study.

The optimal technique and suture material for abdominal wall closure have long been a matter of debate [4]. The ideal suture method should aim at preventing incisional hernia and wound dehiscence without increasing wound infection, wound pain or formation of stitch sinus and Keloid [4, 7]. Despite advances in surgical techniques and materials, abdominal fascia closure has remained a procedure that often reflects a surgeon’s personal preference with reliance on tradition and anecdotal experience [6, 8, 9]. However, generally the selection of a particular suture material is governed by availability, cost and knowledge on sutures.

There are many studies in the literature comparing various methods of wound closure, with conflicting results. Three meta-analyses of these studies have been performed, which have been successful in resolving many of the issues. However, there is still no consensus over continuous versus interrupted methods of wound closure, with one of the meta-analyses favoring the interrupted method [10], another favoring the continuous method and the third not finding any significant difference between the two [11]. In the present study, continuous closure of the fascia was commonly performed in more than ninety percent of cases which is in agreement with other studies [4, 7, 12]. The continuous method of closure has some advantages, namely quick closure with a smaller number of knots, thereby lessening the chances of sinus formation. Because some of the trials have not shown any difference in the complication rates between the two methods, many abdominal surgeons have come to believe in the superiority of continuous closure. The increasing tendency for use of continuous sutures was also reported by several other studies [13]. In keeping with other studies [4, 7, 12, 14], our study showed that continuous suture was significantly associated with low incidence of wound dehiscence and incisional hernia as compared to interrupted suture. This observation conforms with current evidence that shows that using a continuous suture has better results compared with interrupted sutures due to the even distribution of the tension along the entire length of the laparotomy wound [4, 14, 15]. An interrupted technique has the advantage of not being dependent on a single knot compared with the continuous technique; however, it suffers from potential inconsistencies in the tightness of each knot thrown by the surgeon. Inconsistencies in the tension will subject the laparotomy wound to possible tissue ischemia with subsequent necrosis of the wound edge, which in turn can result in wound infection or incisional hernia formation [4, 14]. The lack of an advantage of the interrupted suture in the prevention of incisional hernia probably suggests that incisional hernia results from a multitude of factors and the suturing technique is only one of them. The stretching of the tissues with time, loss of tensile strength of the linea alba and changing dynamics of collagen metabolism with advancing age may play an important role in the pathogenesis of hernia [14].

As reported by other authors [4, 14, 15], mass closure technique in this study was commonly performed and was found to be associated with reduced the time required for closure of the incision, incidence of wound dehiscence and the incidence of incisional hernia. The issue of the mass-closure versus the layered closure of an abdominal wound has been studied in great detail and has produced a high-quality level 1-a evidence of the former’s significant superiority regarding the wound-dehiscence/incisional hernia formation [11].

According to some authors [9, 11], the ideal suture material should have no impact on the incidence of infection, should avoid patient discomfort and should not induce sinus formation in the surgical wound. Studies have compared the use of absorbable versus non-absorbable, delayed-absorbable versus non-absorbable, monofilament versus multifilament for laparotomy closure but without any solid conclusion [4–6, 14]. In the present study, non absorbable sutures (e.g. vicryl) were found to be significantly associated with increased incidence of persistent wound pain and stitch sinus as compared to slowly absorbable sutures and there was no significantly association between non-absorbable sutures and slowly absorbable sutures with respect to incidence of wound dehiscence and incisional hernia. This observation agrees with that reported by Diener et al. [4] in the INLINE systematic review and meta-analysis, but at variant with meta-analysis by Hodgson et al.[12] who reported significantly less incisional hernias after closure with non-absorbable sutures (e.g. prolene and nylon) but also found significantly more suture sinuses and wound pain requiring further interventions. Non absorbable sutures (such as prolene and nylon) are known to be associated with increased incidence of persistence wound pain and stitch sinus; slowly absorbable sutures should be used in preference [7].

Despite lack of statistically significant difference in the current study, the use of multifilament has been reported to be associated with increased incidence of surgical site infections as compared to monofilament. Our finding is in agreement with other authors who reported no association between multifilament suture and surgical site infections [7, 16]. It has been postulated that in multifilament sutures bacteria can escape from phagocytosis in the interstices between the threads and this is probably the reason why monofilament sutures are associated with a lower rate of surgical site infection than multifilament [17, 18].

Findings from this study showed no significantly association between multifilament sutures and monofilament sutures with respect to persistent wound pain and stitch sinus formation. This observation is at variant with other studies that reported significantly association between multifilament sutures and monofilament sutures with respect to these two parameters [18]. We could not establish the reasons for this observation.

As reported by others [7, 16], vicryl was the most commonly used suture material in elective midline laparotomy closure at our centre. However, Rahbari et al. [19] showed the use of slowly absorbable suture like PDSII was higher (55%) followed by moderately absorbable suture like vicryl (39%) and non absorbable suture was used in only 5% of patients. In this study, prolene was significantly associated with persistent wound pain as compared to vickyl. None of our patients in the PDSII group developed any of the complications. However, despite the fact that vickyl is a multifilament suture it was associated with low rate of surgical site infection. The low rate of use of PDSII suture in this study is due to the fact that PDSII is expensive and not readily available.

Exclusion from the study of large number of patients was the potential limitation in this study and might have underestimated our sample size. However, despite this limitation, the study has provided local data that can be utilized by surgeons in our setting to plan for the optimal method of fascial closure following elective midline laparotomy and thus, reduce the incidence of postoperative complications.

## Conclusion

In conclusion continuous mass closure with vickyl suture is commonly used for abdominal fascial closure following elective midline laparotomy at Bugando Medical Centre and it is associated with reduced the time required for closure of the incision, incidence of wound dehiscence and the incidence of incisional hernia as well as persistent wound pain and stitch sinus formation. To reduce the incidence of incisional hernia and wound dehiscence without increasing wound pain or suture sinus frequency, continuous mass closure with vicryl appear to be the optimal method of fascial closure in our setting.
